# Effect of alcohol consumption on kidney function: population-based cohort study

**DOI:** 10.1038/s41598-021-81777-5

**Published:** 2021-01-27

**Authors:** Yu-Ji Lee, Seong Cho, Sung Rok Kim

**Affiliations:** Division of Nephrology, Department of Internal Medicine, Samsung Changwon Hospital, Sungkyunkwan University School of Medicine, 158, Paryong-ro, Masanhoewon-gu, Changwon, 51353 Republic of Korea

**Keywords:** Health care, Nephrology, Risk factors

## Abstract

The association between alcohol consumption and kidney function is intriguing, but study results are mixed and controversial. We examined the association of alcohol consumption with the overall change in kidney function over 12 years. We analyzed data from a population-based cohort that was part of the Korean Genome and Epidemiology Study. Primary exposure was total alcohol intake (non-drinkers, 0 to < 10 g/day, 10 to < 30 g/day, and ≥ 30 g/day). Main outcome was decline in kidney function over 12 years. Our study included 5729 participants (mean [SD] age, 51 [8] years; 46% males). Compared to non-drinkers, higher alcohol intake groups had lesser reduction in estimated glomerular filtration rate (eGFR) over 12 years; fully adjusted beta coefficients and 95% confidence intervals were 0.45 (− 0.27, 1.18), 1.87 (0.88, 2.87), and 3.08 (1.93, 4.24) for participants with alcohol intake of < 10, 10 to < 30, and ≥ 30 g/day, respectively. However, this association was attenuated among women, smoker, and age ≥ 60 year. Compared with not drinking, more frequent alcohol consumption and binge drinking were associated with lesser reduction in eGFR. Our findings suggest that alcohol consumption may have a favorable effect on kidney function among the general population.

## Introduction

Alcohol consumption has been a part of socio-cultural practices worldwide. According to the World Health Organization report in 2016, about 43% of the world’s population over 15 years old reported drinking in the past 12 months^1^. According to the Korean National Health and Nutrition Examination Survey (2013), the drinking rate of men and women was 75.3% and 45.7%, respectively^2^. Alcohol consumption has various effects on health. Although its obvious adverse health effects include liver cirrhosis, cancers, seizure, pancreatitis, poisoning, etc., previous studies have reported that light to moderate alcohol consumption has some beneficial effects such as reduction in the risk of cardiovascular disease and type 2 diabetes^3–7^.

Kidney function, assessed by measurement of the glomerular filtration rate declines by about 8 mL/min/1.73 m^2^ per decade after age 40 years^8^. The decline in kidney function may be accelerated due to various factors such as hypertension, diabetes, primary renal disorders, and some medications causing kidney injury. It is a noteworthy problem that patients with impaired kidney function also consume alcohol. Previous studies have shown that about 20–36% of patients with chronic kidney disease (CKD) consume alcohol either occasionally or daily, and 10% of patients even drink heavily^9–12^. Notwithstanding, the association between alcohol consumption and kidney function has received relatively less attention and studies have been inconclusive. Some studies reported that alcohol consumption was associated with the development or progression of CKD^13^. In other studies, however, alcohol consumption was not associated with kidney function; rather, it was inversely associated with the risk of CKD^14–16^.

Hence, we sought to examine the association of alcohol consumption with the change and rapid decline in kidney function over 12 years in a South Korean population-based cohort study.

## Methods

### Study participants and data collection

We extracted and examined data from a population-based cohort that was part of the Korean Genome and Epidemiology Study (KoGES), a large prospective cohort study. A total of 10,030 participants from the KoGES were recruited in 2001–2002 (baseline). Eligibility criteria included age 40–69 years and residence in Ansan or Ansung, Korea, for at least six months before enrollment. After the baseline examination and survey, biannual follow-up examinations continued through 2014 (comprising six phases of follow-up) (Supplementary Fig. 1). We included participants with baseline data on alcohol consumption and excluded those who did not have data on serum creatinine (Cr) at baseline or at the sixth phase of follow-up. All participants provided written informed consent. The study was approved by the Institutional Review Board of Samsung Changwon Hospital, Sungkyunkwan University School of Medicine (IRB number: SCMC 2020-03-010). All the study methods were performed in accordance with relevant guidelines and regulations of the responsible committee (institutional and national) and with Declaration of Helsinki.

Demographic information on age, sex, comorbid conditions (diabetes, hypertension, hyperlipidemia, and cardio-cerebrovascular disease), household income [< 2,000,000 Korean won (KRW) per month (low), 2,000,000 to < 4,000,000 KRW per month (middle), or ≥ 4,000,000 KRW per month (high)], and education level (middle school or lower, high school, or college or higher), smoking status, height, body weight, systolic blood pressure (SBP), diastolic blood pressure (DBP), nutritional intake (protein, fat, carbohydrate, and sodium), and laboratory variables including serum hemoglobin, albumin, fasting blood sugar (FBS), cholesterol, aspartate aminotransferase (AST), alanine aminotransferase (ALT), serum Cr, C-reactive protein (CRP), and albuminuria were extracted from the database. Albuminuria was defined as a urine albumin-to-Cr ratio ≥ 30 mg/g or a urine protein dipstick test reading ≥ 1 + in the absence of a quantitative test result.

### Assessment of alcohol consumption

Participants were surveyed on their alcohol consumption habits during the preceding year. Information was collected on the frequency of drinking alcoholic beverages including unrefined or refined rice wine, beer, Korean distilled liquor, wine and liquors; amount per serving (glass); and the size of the drinking glass. The amount of pure alcohol in grams per day was calculated based on the frequency of alcohol consumption by alcohol type over the past year, the amount of each alcohol drink per one drinking occasion, the alcohol content of each alcoholic beverage (4.5% for beer, 13% for wine, 40% for liquor, 22% for Korean distilled liquor, 15% for refined rice wine, and 6% for unrefined rice wine), and ethanol density (0.789 g/mL). Total alcohol intake (in grams per day) was calculated as the sum of the amount of pure alcohol consumed per day in the form of the above beverages. Participants were categorized into four groups according to total alcohol intake: 0 g/day (non-drinkers), 0 to < 10 g/day, 10 to < 30 g/day, and ≥ 30 g/day. Based on the weekly frequency of alcohol consumption, participants were classified into four groups: non-drinkers, < 1 times/week, 1–3 times/week, and ≥ 4 times/week. Binge drinking was defined as consuming, on average, seven or more drinks at a time for men or five or more drinks at a time for women.

### Exposures and outcomes

The primary exposure was baseline total alcohol intake divided into four categories. The primary outcome was a decline in kidney function over 12 years. This was assessed by measuring the change in the estimated glomerular filtration rate (eGFR) calculated by subtracting the baseline eGFR from the eGFR at the sixth phase of follow-up. A negative value of the change in eGFR indicates a fall in eGFR. The association of the secondary exposures—frequency of alcohol consumption and binge drinking—with the change in the eGFR were also assessed. The secondary outcome was a rapid decline in kidney function, defined as a decrease in the eGFR ≥ 20 mL/min/1.73 m^2^ over 12 years. The eGFR was calculated according to the CKD-EPI (Chronic Kidney Disease Epidemiology Collaboration) equation^17^.

### Statistical analyses

The significance of trends in variables across total alcohol intake categories was determined using the Wilcoxon-type nonparametric trend test or a linear regression analysis, as appropriate. To assess the association between categorized alcohol intake and change in the eGFR, linear regression analysis was performed using non-drinkers as the reference. Three hierarchical adjustment models were used: (1) an unadjusted model (model 1); (2) a case mix-adjusted model (model 2) that included age, sex, comorbidities (diabetes, hypertension, hyperlipidemia, and cardio-cerebrovascular disease), body mass index, household income (low, middle, or high), education level (middle school or lower, high school, or college or higher), smoking status (current smoking or non-smoking), SBP, DBP, and nutritional intake (protein, fat, carbohydrate, and sodium); and (3) a fully adjusted model (model 3) that included eGFR, FBS, cholesterol, hemoglobin, AST, ALT, CRP, and albuminuria in addition to all of the above variables. The association of the frequency of alcohol consumption and binge drinking with change in the eGFR was assessed using a linear regression model with the aforementioned levels of adjustment.

To examine effect modification by age, sex, diabetes, hypertension, smoking status, baseline eGFR, and albuminuria on the association between total alcohol intake and change in the eGFR, a likelihood ratio test was performed by adding an interaction term between alcohol intake and each of the above covariates to the linear regression model, followed by subgroup analyses according to age (< 60 or ≥ 60 years), sex, diabetes, hypertension, smoking status, baseline eGFR (< 90 or ≥ 90 mL/min/1.73 m^2^), and albuminuria.

Considering the sick-quitter effect, defined as not drinking alcohol due to existing health conditions, regression analysis was performed after excluding non-drinkers as a sensitivity analysis. In addition, a sensitivity analysis was performed with further adjustments for variables on health conditions that could affect alcohol consumption: the current or past history of cancer, continuous medication use for the past three months or more, and self-rated health (“very good or good”, “fair”, and “bad or very bad”). The rate of decline in kidney function over 12 years was estimated in relation to the baseline alcohol intake categories. A linear mixed-effects model was used that allowed for a random intercept and slope using an unstructured covariance matrix.

Missing values were found for some covariates including diabetes, hypertension, hyperlipidemia, education, income, nutritional intake, body mass index, smoking status, SBP, DBP, FBS, cholesterol, hemoglobin, AST, ALT, CRP, and albuminuria. The frequency of missing data was < 0.2% for all covariates except smoking status (0.7%), education (0.5%), income (1.5%), nutritional intake (2.2%), and FBS (2%). Mean or median imputation methods were performed to treat these missing data. All analyses were performed using STATA, version 14.2 (StataCorp LP, College Station, TX, USA).

## Results

### Participant characteristics

Data from 10,030 participants were extracted from a de-identified dataset for analysis, and 9724 participants who had baseline data on alcohol consumption were included in our cohort. After excluding participants without data on serum Cr at baseline and the sixth phase of follow-up, data from 5729 participants were available for analysis. A flow diagram summarizes cohort construction (Supplementary Fig. 2).

Of the total participants, 2670 were current alcohol drinkers, while 3059 were either non-drinkers from the beginning (n = 2726) or former drinkers (n = 333). The baseline characteristics of the participants across total alcohol intake categories are shown in Table 1. In our cohort, the mean ± SD age of the participants was 51 ± 8 years and 46% were men. Fifty-three percent of the participants reported not drinking alcohol during the past month. The median (IQR) alcohol consumed by drinkers was 8.9 (2.7, 26.1) g/day. Groups with higher alcohol intake had a higher proportion of men and smokers and tended to have lower baseline eGFR and higher median values of FBS, cholesterol, AST, ALT, and CRP. The proportion of participants with greater household income and higher education levels was higher in groups with larger alcohol consumption. The intake of protein, fat, carbohydrate, and sodium was also higher in groups with larger alcohol consumption. The trajectory of total alcohol intake over 12 years showed a consistent difference across baseline alcohol intake categories (Supplementary Fig. 3).

**Table 1:** Baseline characteristics of 5729 participants according to groups defined by baseline alcohol consumption.

Patient characteristics	Total	Baseline alcohol consumption category				P value
		Group 1 Non-drinkers	Group 2 < 10 g/day	Group 3 10–30 g/day	Group 4 ≥ 30 g/day	
Total population, N (%)	5729	3059 (53)	1372 (24)	758 (13)	540 (10)	
Age, years	51 ± 8	52 ± 9	50 ± 8	50 ± 8	50 ± 8	< 0.001
Male, %	2638 (46)	745 (24)	692 (50)	681 (90)	520 (96)	< 0.001
Current smoker, %	1285 (23)	304 (10)	322 (24)	369 (49)	290 (54)	< 0.001
Body mass index, kg/m^2^	24.7 ± 3.1	24.8 ± 3.2	24.6 ± 3.0	24.6 ± 2.8	24.7 ± 3.0	0.917
**Comorbidities, %**						
Diabetes mellitus	735 (13)	412 (13)	128 (9)	102 (13)	93 (17)	0.196
Hypertension	1877 (33)	1023 (33)	354 (26)	286 (38)	214 (40)	0.01
Hyperlipidemia	158 (3)	66 (2)	38 (3)	34 (4)	20 (4)	0.001
Cardiovascular disease	125 (2)	76 (2)	22 (2)	14 (2)	13 (2)	0.397
**Household income**^**a**^**, %**						< 0.001
Low	3483 (62)	2047 (68)	799 (59)	400 (53)	237 (44)	
Middle	1678 (30)	751 (25)	455 (33)	268 (36)	204 (38)	
High	485 (9)	197 (7)	107 (8)	86 (11)	95 (18)	
**Education level, %**						< 0.001
Middle school or lower	3001 (53)	1871 (62)	634 (46)	284 (37)	212 (39)	
High school	1881 (33)	860 (28)	510 (37)	295 (39)	216 (40)	
College or higher	816 (14)	302 (10)	224 (16)	179 (24)	111 (21)	
**Alcohol consumption**						
Frequency, times/month	1 (0–2)	0 (0–0)	2 (1–2)	2 (2–3)	3 (2–3)	< 0.001
Amount per occasion, glass/occasion	1 (0–6)	0 (0–0)	3 (2–6)	7 (5–10)	10 (7–14)	< 0.001
Total alcohol intake, g/day	0.5 (0.0–11.6)	0.0 (0.0–0.0)	4.2 (2.1–7.9)	19.6 (14.2–27.0)	47.1 (34.7–66.0)	< 0.001
Systolic blood pressure, mmHg	120 ± 18	120 ± 19	118 ± 18	121 ± 18	123 ± 18	< 0.001
Diastolic blood pressure, mmHg	80 ± 12	79 ± 12	79 ± 12	82 ± 11	83 ± 12	< 0.001
**Nutritional intake, g/day**						
Protein	62 (48–78)	59 (45–75)	63 (50–79)	65 (50–82)	71 (56–87)	< 0.001
Fat	29 (19–41)	26 (17–37)	31 (21–41)	33 (22–46)	37 (25–50)	< 0.001
Carbohydrate	325 (279–385)	323 (276–387)	326 (281–381)	323 (286–374)	334 (289–390)	0.009
Sodium	2.9 (2.1–3.9)	2.8 (2.0–3.7)	2.9 (2.1–3.9)	3.1 (2.3–4.1)	3.3 (2.5–4.6)	< 0.001
**Laboratory variables**						
Hemoglobin, g/dL	13.6 ± 1.6	13.1 ± 1.5	13.6 ± 1.6	14.6 ± 1.3	14.8 ± 1.2	< 0.001
Serum albumin, g/dL	4.5 (4.3–4.7)	4.5 (4.3–4.6)	4.5 (4.3–4.7)	4.6 (4.4–4.8)	4.6 (4.4–4.8)	< 0.001
Fasting blood sugar, mg/dL	88 (82–94)	87 (82–93)	87 (82–92)	90 (85–98)	92 (86–101)	< 0.001
Total cholesterol, mg/dL	189 (168–213)	188 (167–211)	188 (167–212)	191 (170–215)	195 (170–220)	< 0.001
AST, IU/L	22 (18–28)	21 (18–26)	22 (18–27)	25 (21–31)	27 (22–33)	< 0.001
ALT, IU/L	19 (14–27)	17 (13–24)	19 (14–27)	23 (18–33)	26 (19–34)	< 0.001
Serum creatinine, mg/dL	0.8 (0.7–0.9)	0.7 (0.7–0.9)	0.8 (0.7–1.0)	0.9 (0.8–1.0)	1.0 (0.8–1.1)	< 0.001
eGFR, mL/min/1.73 m^2^	95 (83–104)	96 (85–104)	96 (83–105)	93 (82–103)	92 (82–102)	0.034
C-reactive protein, mg/L	0.1 (0.1–0.2)	0.1 (0.1–0.2)	0.1 (0.1–0.2)	0.1 (0.1–0.2)	0.2 (0.1–0.3)	0.021
Albuminuria, %	341 (6)	204 (7)	69 (5)	35 (5)	33 (6)	0.09

*AST* aspartate aminotransferase, *ALT* alanine aminotransferase, *eGFR* estimated glomerular filtration rate.

SI conversion factor: to convert hemoglobin from g/dL to g/L multiply by 10; to convert serum albumin in g/dL to g/L, multiply by 10; to convert creatinine to μmol/L, multiply by 88.4; to convert total cholesterol from mg/dL to mmol/L multiply by 0.0259.

Values for categorical variables are shown as No. (%); values for continuous variables, as mean ± standard deviation or median (interquartile range).

^a^Household income was categorized into three groups; < 2,000,000 Korean won (KRW) per month (low), 2,000,000 to < 4,000,000 KRW per month (middle), and ≥ 4,000,000 KRW per month (high).

### Association between total alcohol intake and change in the eGFR over 12 years

**Figure 1:**
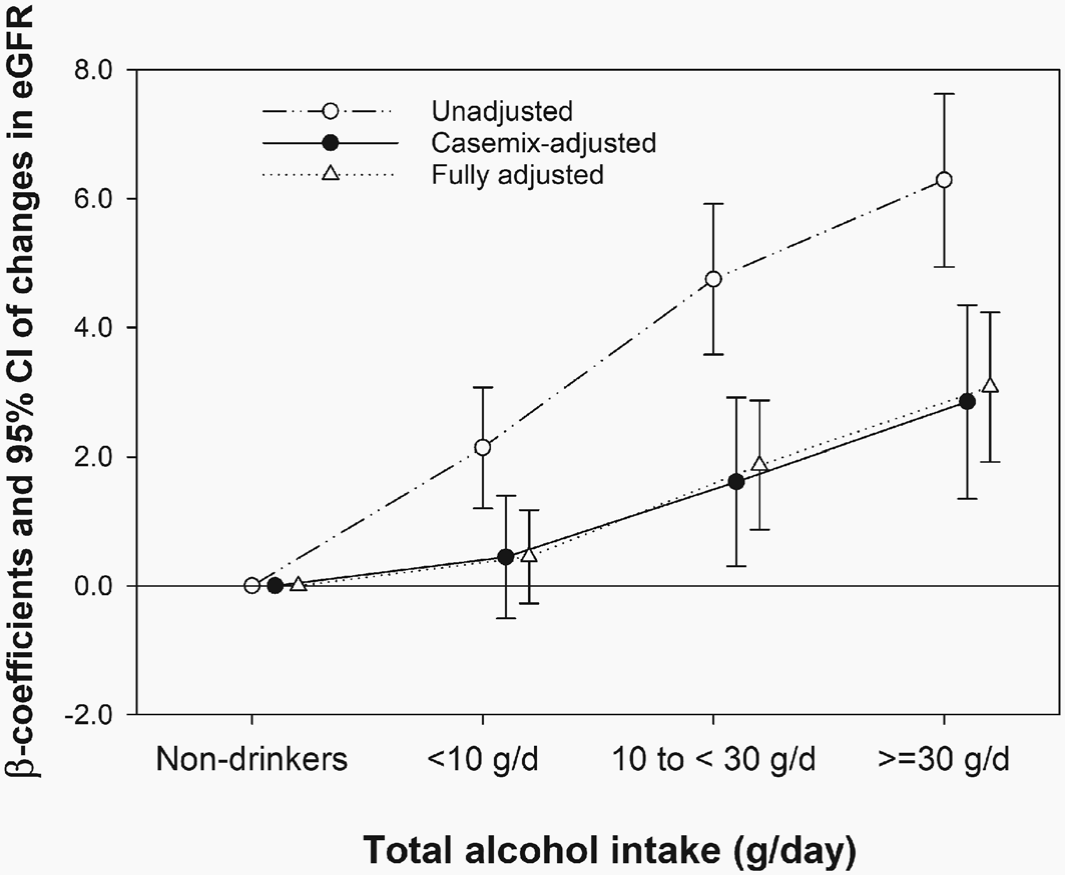
The association of baseline alcohol consumption and decline in kidney function over 12 years among 5729 participants. Points and bars represent beta coefficients and 95% confidence intervals, respectively.

The median (IQR) change in the eGFR over 12 years was − 23.6 (− 32.6, − 11.6) mL/min/1.73 m^2^. In linear regression analyses, the amount of alcohol consumption was inversely associated with decline in kidney function. Compared with the reference group (non-drinkers), higher alcohol intake groups had lesser decline in the eGFR over 12 years; fully adjusted beta coefficients (β) and 95% confidence intervals (CIs) were 0.45 (− 0.27, 1.18), 1.87 (0.88, 2.87), and 3.08 (1.93, 4.24) for the groups with baseline total alcohol intake of < 10 g/day, 10 to < 30 g/day, and ≥ 30 g/day, respectively (Fig. 1).

**Figure 2:**
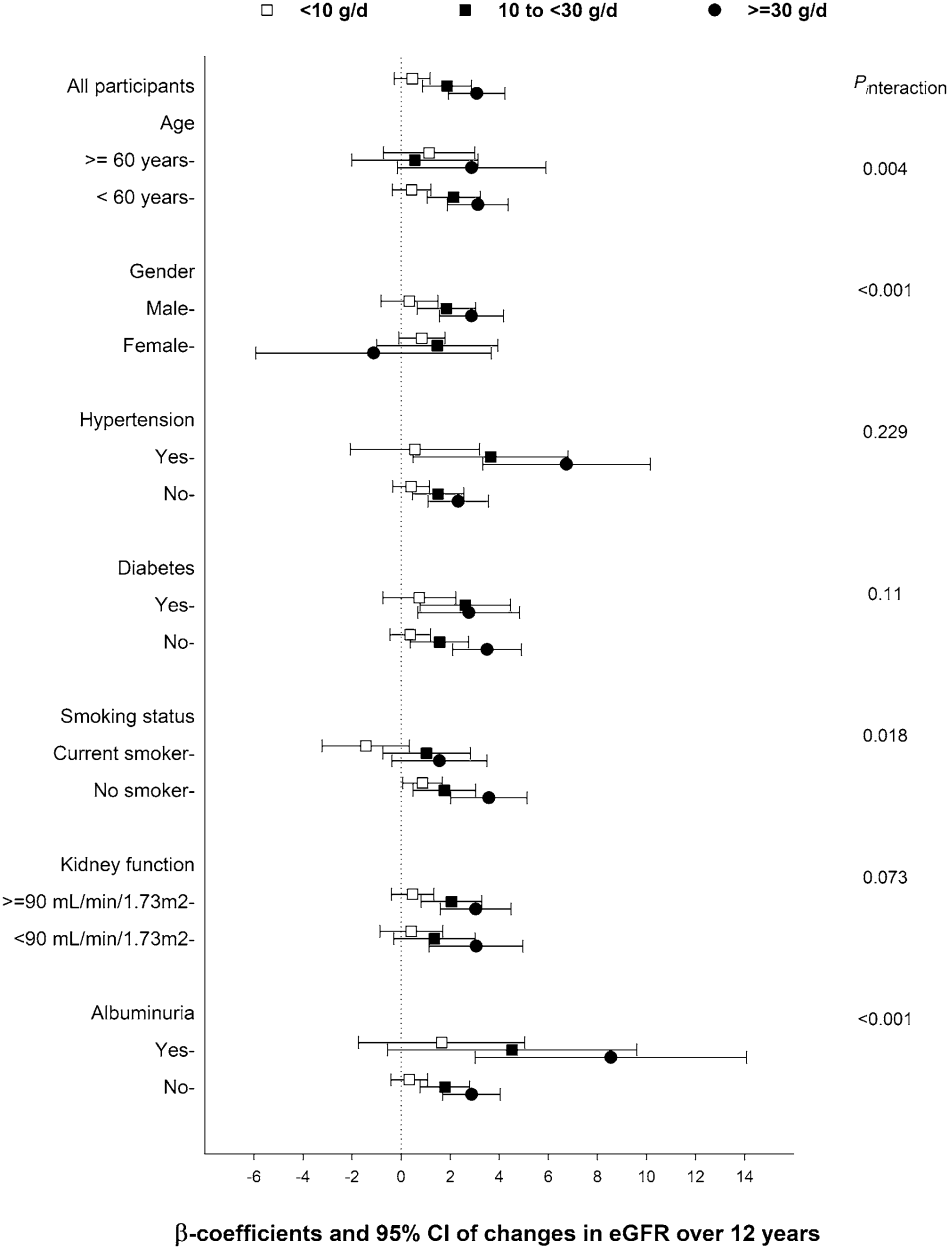
Overall and subgroup analyses of the association between baseline alcohol consumption and decline in kidney function over 12 years in fully adjusted linear regression model. Point and bars represent beta coefficients and 95% confidence intervals, respectively. The reference group consists of non-drinkers.

In subgroup analyses, the association between total alcohol intake and change in the eGFR was modified by sex (*P*_interaction_ < 0.001), smoking status (*P*_interaction_ = 0.018), age (*P*_interaction_ = 0.004), and albuminuria (*P*_interaction_ < 0.001). The inverse association between total alcohol intake and decline in kidney function was attenuated among women, smoker, and age ≥ 60 years (Fig. 2). An inverse association between total alcohol intake and change in the eGFR was found among participants both with and without albuminuria, although it was more pronounced among those with albuminuria. However, diabetes (*P*_interaction_ = 0.11), hypertension (*P*_interaction_ = 0.229), and baseline eGFR (*P*_interaction_ = 0.073) did not modify the association of alcohol intake with change in the eGFR.

**Figure 3:**
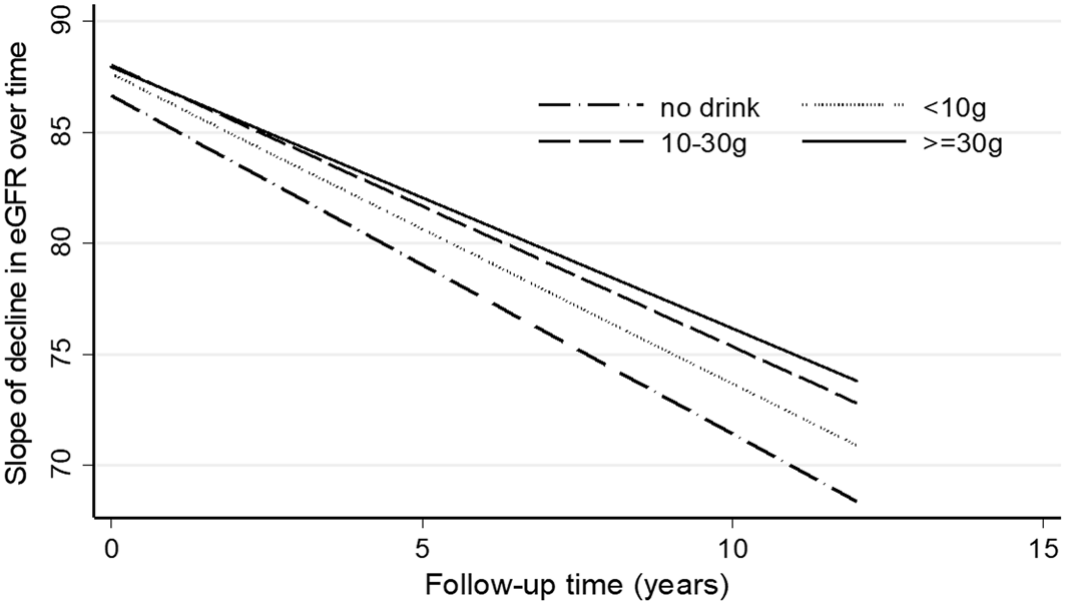
The slope of the estimated glomerular filtration rate (eGFR) over 12 years according to baseline alcohol consumption categories among 5729 participants.

The slope of decline in the eGFR estimated over 12 years was less steep in the higher alcohol intake groups (Fig. 3).

### Sensitivity analysis: the association between total alcohol intake and change in the eGFR among current alcohol drinkers

Total alcohol intake in the 2,670 current drinkers was reclassified into five categories: < 5 g/day (n = 985), 5 to < 20 g/day (n = 777), 20 to < 35 g/day (n = 451), 35 to < 50 g/day (n = 197), and ≥ 50 g/day (n = 260). The higher alcohol intake groups had lesser decline in kidney function than the lowest alcohol intake group (reference: < 5 g/day): the fully adjusted β and 95% CIs for change in the eGFR were 0.49 (− 0.65, 1.63), 1.69 (0.31, 3.06), 2.39 (0.57, 4.20), and 1.95 (0.26, 3.63) for the groups with total alcohol intake of 5 to < 20 g/day, 20 to < 35 g/day, 35 to < 50 g/day, and ≥ 50 g/day, respectively.

### Sensitivity analysis: further adjustment for other variables on health conditions that could affect alcohol consumption

Compared to drinkers, non-drinkers were more likely to have the past or current history of cancer (2.9% vs 1.9%), to use medication for the past three months or more (43.0% vs 32.8%), and to rate themselves unhealthy (37.0% vs 23.4%). However, in sensitivity analysis with additional adjustment for these variables, the results remained consistent; fully adjusted β and 95% CIs were 0.46 (− 0.26, 1.19), 1.86 (0.86, 2.86), and 3.05 (1.89, 4.21) for the groups with baseline total alcohol intake of < 10 g/day, 10 to < 30 g/day, and ≥ 30 g/day, respectively (reference: non-drinkers).

### Association of monthly frequency of alcohol consumption and binge drinking with change in the eGFR over 12 years

Of the 2670 current drinkers, 949, 1175, and 546 drank alcohol at a frequency of < 1 times per week, 1–3 times per week, and ≥ 4 times per week, respectively. In addition, 1064 were binge drinkers.

In the linear regression analysis, more frequent alcohol consumption was associated with lesser reduction in eGFR over 12 years compared to not drinking; the fully adjusted β and 95% CIs were 0.28 (− 0.54, 1.09), 1.89 (1.04, 2.74), and 1.83 (0.70, 2.96) for groups with a frequency of alcohol consumption of < 1 times per week, 1–3 times per week, and ≥ 4 times per week, respectively (reference: non-drinkers).

Binge drinking was also associated with lesser reduction in eGFR over 12 years compared to not drinking. The fully adjusted β and 95% CIs for change in the eGFR were 0.66 (− 0.05, 1.36) and 2.24 (1.33, 3.15) for light to moderate drinkers and binge drinkers, respectively (reference: non-drinkers).

### Association between total alcohol intake and rapid decline in kidney function

**Figure 4:**
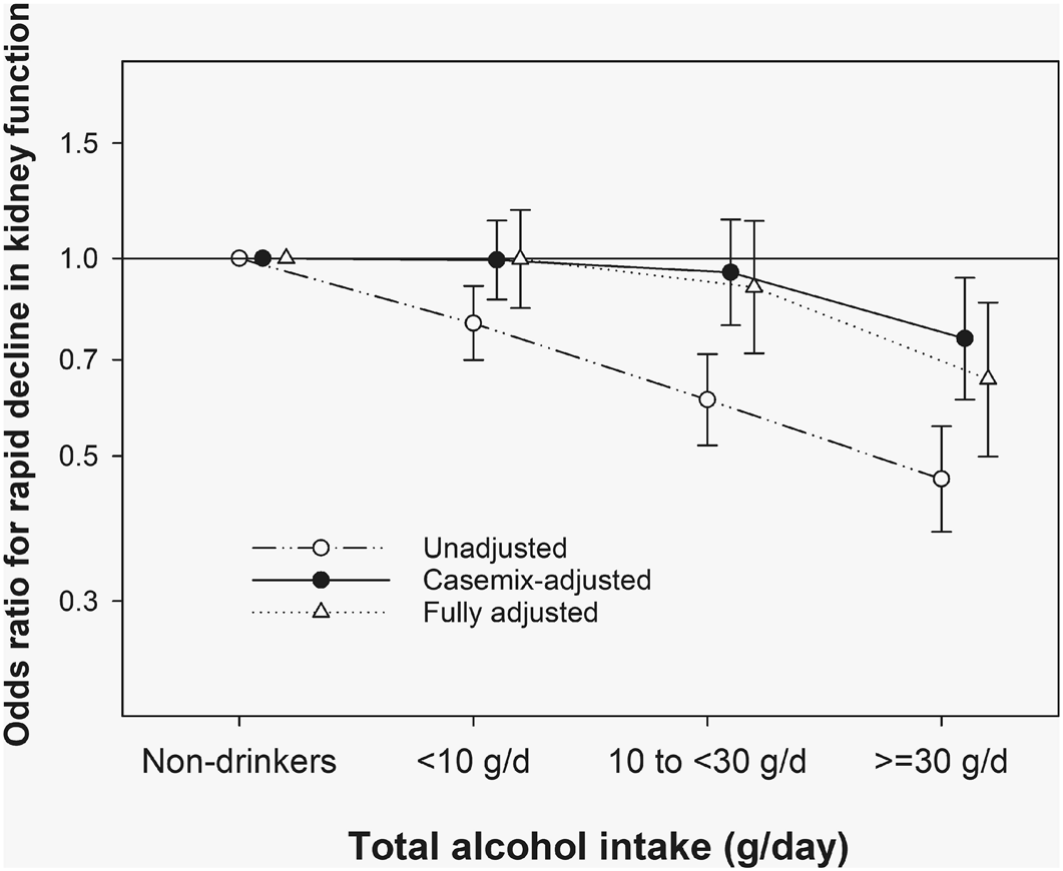
Association between total alcohol intake and rapid decline in kidney function (odds ratios) with three levels of adjustment in relation to baseline alcohol consumption among 5729 participants.

In 3,407 (59.5%) participants, the eGFR decreased by more than 20 mL/min/1.73 m^2^ over 12 years. In the logistic regression analysis, the highest alcohol intake group had 37% lower odds of rapid decline in kidney function compared with non-drinkers; the fully adjusted odds ratios and 95% CIs were 1.0 (0.84, 1.19), 0.90 (0.72, 1.14), and 0.65 (0.50, 0.86) for those with a baseline total alcohol intake of < 10 g/day, 10 to < 30 g/day, and ≥ 30 g/day, respectively (Fig. 4).

## Discussion

In this population-based cohort study, we found that alcohol consumption was negatively associated with decline in kidney function in general population, independently of baseline kidney function and comorbidities. Higher total alcohol intake, more frequent alcohol consumption, and binge drinking were associated with a lesser decline in the eGFR. This association was more pronounced among non-smokers, participants aged under 60 years, and those with albuminuria; however, it was completely attenuated among women.

Alcohol consumption has known to be associated with health problems including liver disease, pancreatitis, neurologic complications, and certain cancers^3–5^. This common knowledge may lead to the hasty assumption that alcohol consumption may also cause kidney damage and consequently contribute to a decline in kidney function. Animal studies have shown that alcohol may cause kidney injury by inducing oxidative stress and inflammation^18,19^. Some population-based studies have also suggested that alcohol consumption may increase the risk of CKD or end-stage renal disease in the general population^20,21^. However, a study by Yuan et al. found that alcohol preserved renal function by reducing tubular damage and inflammation in mice with renal ischemic/reperfusion injury^22^. Other studies have also suggested an inverse association between alcohol consumption and the risk of renal impairment or CKD^11,14,15^. In concordance with the latter studies, our study showed that higher alcohol consumption was associated with a slower decline in kidney function in terms of both the amount consumed and the frequency of consumption.

There are several potential mechanisms to explain the inverse association between alcohol consumption and decline in kidney function. First of all, polyphenolic compounds in alcoholic beverages exhibit antioxidant and anti-inflammatory properties, both of which may have renal protective effects^23–25^. Among patients with type 2 diabetes, resveratrol, a polyphenolic compound, was found to reduce the levels of serum Cr, urea nitrogen, and total cholesterol, suggesting improved kidney function and lipid profile^26,27^. Moreover, even polyphenol-free alcoholic beverages have been found to exert anti-inflammatory or antioxidant effects, even though they were less effective than those with high amounts of polyphenol^28–30^. Second, alcohol consumption is associated with an increase in insulin sensitivity^31,32^. Given that insulin resistance and concomitant hyperinsulinemia are associated with renal dysfunction in the general population^33,34^, the improvement of insulin sensitivity due to alcohol consumption may have a beneficial effect on kidney function.

In our study, the favorable effect of alcohol consumption on kidney function was observed among only men. Sex differences in the effects of alcohol consumption were also reported in some other studies^11,35^. Although the plausible biological mechanism responsible for these differences is unclear, it can be explained in part by oxidative stress, which is known to be enhanced in men compared with women^36^. Moreover, the renal markers of oxidative stress were significantly elevated in male rats compared with female rats in animal studies^37,38^. Long-term exposure to red wine with high polyphenol contents was shown to have antioxidant effects on rat kidneys^39^. Therefore, the antioxidant effects of alcoholic beverages on kidney may be more pronounced among men. We also found that the inverse association between alcohol consumption and kidney function was pronounced among non-smokers and younger people (< 60 years). Smoking and aging are associated with oxidative stress, and consequently, with a decline in kidney function^40–43^. Therefore, they may attenuate the beneficial effects of alcohol on kidney function.

The strength of our study is that it conducted comprehensive analyses on several aspects of decline in kidney function with regard to drinking patterns, including the amount and frequency of alcohol consumption. However, our study has several limitations to be acknowledged. First, because of the observational nature of our study, there may be unmeasured confounding even though the models were adjusted for potential confounding factors. In particular, the possibility that non-drinkers at baseline were sick quitters who quit alcohol consumption due to other illnesses cannot be ruled out. However, the inverse association between alcohol consumption and kidney function remained robust after excluding those who did not consume alcohol at baseline. Furthermore, the association remained consistent even after adjusting for other variables on health condition who could affect alcohol consumption. Second, the amount and/or frequency of alcohol consumption may change over time. However, based on previous cohort studies, alcohol consumption in the general population was found to remain generally stable within the baseline-defined drinking categories during the course of their lives^44^. In our study cohort, the trajectories of alcohol consumption also maintained similarities to the baseline levels over 12 years. In addition, when the analysis was repeated only in participants assigned to the same alcohol intake category as that at baseline at the last follow-up, the results remained consistent (Supplementary Fig. 4). Third, although participants were asked questions regarding their alcohol consumption habits by trained interviewers, the amount or frequency of alcohol consumption may have been under-reported due to negative sociocultural perception about heavy drinking. However, given that more alcohol consumption appears to be associated with better preservation of kidney function, this would have likely biased our results towards the null. Fourth, the median baseline eGFR of our cohort was 95 mL/min/1.73 m^2^. Only 88 participants had moderate to severe kidney dysfunction at baseline. Therefore, our study cannot determine whether the inverse association between alcohol consumption and kidney function is also valid among patients with CKD stage 3–5. Finally, our study included only patients with data on serum Cr at baseline and at the sixth phase of follow-up, which could have led to selection bias. However, there were no significant differences in baseline characteristics between included and excluded participants (Supplementary Table 1).

In conclusion, more alcohol consumption was associated with lesser decline in kidney function over 12 years among the general population in Korea, especially men. Although we found a favorable effect of alcohol consumption on kidney function, our results should not be used to encourage or justify excessive alcohol consumption for kidney health because of many other known health or social problems associated with drinking, especially excessive drinking. However, given our results, there is a lack of grounds that alcohol consumption should be discouraged just for kidney health. Considering both the beneficial and detrimental effects of alcohol consumption, additional studies are needed to determine the appropriate amount of alcohol consumption. Moreover, the effects of alcohol consumption among patients with moderate to severe renal impairment need to be studied further.

## Supplementary Information

Supplementary Information.

## Data Availability

The database is publicly available at the Korea Center for Disease Control website (http://is.cdc.go.kr/).

## References

[1] (WHO), W. H. O. Global Status Report on Alcohol and Health 2018. (World Health Orgaization, Geneva, 2018).

[2] Health, K. N. I. o. Annual Report 2016. (2016).

[3] Mandayam S, Jamal MM, Morgan TR (2004). Epidemiology of alcoholic liver disease. Semin. Liver Dis..

[4] Rocco A, Compare D, Angrisani D, Sanduzzi Zamparelli M, Nardone G (2014). Alcoholic disease: Liver and beyond. World J. Gastroenterol..

[5] Bagnardi V (2015). Alcohol consumption and site-specific cancer risk: A comprehensive dose-response meta-analysis. Br. J. Cancer.

[6] Ronksley PE, Brien SE, Turner BJ, Mukamal KJ, Ghali WA (2011). Association of alcohol consumption with selected cardiovascular disease outcomes: A systematic review and meta-analysis. BMJ.

[7] Baliunas DO (2009). Alcohol as a risk factor for type 2 diabetes: A systematic review and meta-analysis. Diabetes Care.

[8] Weinstein JR, Anderson S (2010). The aging kidney: Physiological changes. Adv. Chron. Kidney Dis..

[9] Bundy JD (2018). Self-reported tobacco, alcohol, and illicit drug use and progression of chronic kidney disease. Clin. J. Am. Soc. Nephrol..

[10] Chen J (2017). Association between metabolic syndrome and chronic kidney disease in a Chinese urban population. Clin. Chim. Acta.

[11] Hsu YH, Pai HC, Chang YM, Liu WH, Hsu CC (2013). Alcohol consumption is inversely associated with stage 3 chronic kidney disease in middle-aged Taiwanese men. BMC Nephrol..

[12] Shimizu Y (2011). Chronic kidney disease and drinking status in relation to risks of stroke and its subtypes: The Circulatory Risk in Communities Study (CIRCS). Stroke.

[13] Joo YS (2020). Alcohol consumption and progression of chronic kidney disease: Results from the korean cohort study for outcome in patients with chronic kidney disease. Mayo Clin. Proc..

[14] Schaeffner ES (2005). Alcohol consumption and the risk of renal dysfunction in apparently healthy men. Arch. Intern. Med..

[15] Koning SH (2015). Alcohol consumption is inversely associated with the risk of developing chronic kidney disease. Kidney Int..

[16] White SL (2009). Alcohol consumption and 5-year onset of chronic kidney disease: The AusDiab study. Nephrol. Dial. Transplant..

[17] Levey AS (2009). A new equation to estimate glomerular filtration rate. Ann. Intern. Med..

[18] Latchoumycandane C, Nagy LE, McIntyre TM (2014). Chronic ethanol ingestion induces oxidative kidney injury through taurine-inhibitable inflammation. Free Radic. Biol. Med..

[19] Harris PS (2015). Chronic ethanol consumption induces mitochondrial protein acetylation and oxidative stress in the kidney. Redox Biol..

[20] Shankar A, Klein R, Klein BE (2006). The association among smoking, heavy drinking, and chronic kidney disease. Am. J. Epidemiol..

[21] Perneger TV, Whelton PK, Puddey IB, Klag MJ (1999). Risk of end-stage renal disease associated with alcohol consumption. Am. J. Epidemiol..

[22] Yuan Q (2011). Preconditioning with physiological levels of ethanol protect kidney against ischemia/reperfusion injury by modulating oxidative stress. PLoS ONE.

[23] Drel VR, Sybirna N (2010). Protective effects of polyphenolics in red wine on diabetes associated oxidative/nitrative stress in streptozotocin-diabetic rats. Cell Biol. Int..

[24] Nakamura T, Fujiwara N, Sugaya T, Ueda Y, Koide H (2009). Effect of red wine on urinary protein, 8-hydroxydeoxyguanosine, and liver-type fatty acid-binding protein excretion in patients with diabetic nephropathy. Metabolism.

[25] Yahfoufi N, Alsadi N, Jambi M, Matar C (2018). The immunomodulatory and anti-inflammatory role of polyphenols. Nutrients..

[26] Bhatt JK, Thomas S, Nanjan MJ (2012). Resveratrol supplementation improves glycemic control in type 2 diabetes mellitus. Nutr. Res..

[27] Den Hartogh DJ, Tsiani E (2019). Health benefits of resveratrol in kidney disease: Evidence from in vitro and in vivo studies. Nutrients..

[28] Estruch R (2004). Different effects of red wine and gin consumption on inflammatory biomarkers of atherosclerosis: A prospective randomized crossover trial. Effects of wine on inflammatory markers. Atherosclerosis.

[29] Badia E (2004). Decreased tumor necrosis factor-induced adhesion of human monocytes to endothelial cells after moderate alcohol consumption. Am. J. Clin. Nutr..

[30] Estruch R (2011). Moderate consumption of red wine, but not gin, decreases erythrocyte superoxide dismutase activity: A randomised cross-over trial. Nutr. Metab. Cardiovasc. Dis..

[31] Davies MJ (2002). Effects of moderate alcohol intake on fasting insulin and glucose concentrations and insulin sensitivity in postmenopausal women: A randomized controlled trial. JAMA.

[32] Joosten MM, Beulens JW, Kersten S, Hendriks HF (2008). Moderate alcohol consumption increases insulin sensitivity and ADIPOQ expression in postmenopausal women: A randomised, crossover trial. Diabetologia.

[33] Chen J (2003). Insulin resistance and risk of chronic kidney disease in nondiabetic US adults. J. Am. Soc. Nephrol..

[34] Kubo M (1999). Effect of hyperinsulinemia on renal function in a general Japanese population: The Hisayama study. Kidney Int..

[35] Buja A (2011). Renal impairment and moderate alcohol consumption in the elderly. Results from the Italian Longitudinal Study on Aging (ILSA). Public Health Nutr..

[36] Ide T (2002). Greater oxidative stress in healthy young men compared with premenopausal women. Arterioscler. Thromb. Vasc. Biol..

[37] Ojeda NB (2012). Oxidative stress contributes to sex differences in blood pressure in adult growth-restricted offspring. Hypertension.

[38] Wang X, Desai K, Juurlink BH, de Champlain J, Wu L (2006). Gender-related differences in advanced glycation endproducts, oxidative stress markers and nitric oxide synthases in rats. Kidney Int..

[39] Rodrigo R, Rivera G, Orellana M, Araya J, Bosco C (2002). Rat kidney antioxidant response to long-term exposure to flavonol rich red wine. Life Sci..

[40] Mayyas F, Alzoubi KH (2019). Impact of cigarette smoking on kidney inflammation and fibrosis in diabetic rats. Inhal. Toxicol..

[41] Arany I, Taylor M, Fulop T, Dixit M (2018). Adverse effects of chronic nicotine exposure on the kidney: Potential human health implications of experimental findings. Int. J. Clin. Pharmacol. Ther..

[42] Csiszar A, Toth J, Peti-Peterdi J, Ungvari Z (2007). The aging kidney: Role of endothelial oxidative stress and inflammation. Acta Physiol. Hung..

[43] Finkel T, Holbrook NJ (2000). Oxidants, oxidative stress and the biology of ageing. Nature.

[44] Knott CS, Bell S, Britton A (2018). The stability of baseline-defined categories of alcohol consumption during the adult life-course: A 28-year prospective cohort study. Addiction.

