# Supplementary material for: Effect of alcohol consumption on kidney function: population-based cohort study
Source: Sci Rep. 2021 Jan 27;11:2381. doi: 10.1038/s41598-021-81777-5 (PMC7840672; doi:10.1038/s41598-021-81777-5)
Supplement: Supplementary file 1 — Supplementary Information. [file 41598_2021_81777_MOESM1_ESM.docx]

**Effect of Alcohol Consumption on Kidney Function: Population-based Cohort Study**

(Running title: Alcohol and Kidney Function)

Yu-Ji Lee^*^, Seong Cho, and Sung Rok Kim

Affiliations

Division of Nephrology, Department of Internal Medicine, Samsung Changwon Hospital, Sungkyunkwan University School of Medicine, Changwon, Korea

**Supplemental material**

Supplemental Table 1. Comparison of baseline characteristics between included and excluded participants.

Supplemental Figure 1. Flow diagram of baseline recruitment and follow-up for the Korean genome and epidemiology study (KoGES).

Supplemental Figure 2. Cohort construction

Supplemental Figure 3. 12-year trajectories of total alcohol intake according to baseline alcohol consumption categories among 5,729 participants.

Supplemental Figure 4. The association of alcohol consumption with decline in kidney function over 12 years among 3,734 participants assigned to the same alcohol intake category as that at baseline at the last follow-up. Points and bars represent beta coefficients and 95% confidence intervals, respectively.

**Supplemental Table 1. Comparison of baseline characteristics between included and excluded participants.**

| Variables | All participants 9,724 | Included 5,729 (59%) | Excluded 3,995 (41%) | Standardized difference^*^ |
| --- | --- | --- | --- | --- |
| Age, years | 52 ± 9 | 51 ± 8 | 54 ± 10 | 0.15 |
| Man, % | 47 | 46 | 49 | 0.06 |
| Current smoker, % | 25 | 23 | 30 | 0.16 |
| Body mass index, kg/m^2^ | 24.6 ± 3.1 | 24.7 ± 3.1 | 24.4 ± 3.2 | -0.05 |
| Comorbidities, % |  |  |  |  |
| Diabetes mellitus | 15 | 13 | 18 | 0.16 |
| Hypertension | 35 | 33 | 38 | 0.11 |
| Hyperlipidemia | 2 | 3 | 2 | -0.05 |
| Cardiovascular disease | 3 | 2 | 4 | 0.10 |
| Household income^**^, % |  |  |  |  |
| Low | 65 | 62 | 69 | 0.16 |
| Middle | 28 | 30 | 25 | -0.11 |
| High | 8 | 9 | 6 | -0.10 |
| Education level, % |  |  |  |  |
| Middle school or lower | 56 | 53 | 62 | 0.18 |
| High school | 30 | 33 | 26 | -0.14 |
| College or higher | 13 | 14 | 12 | -0.07 |
| Alcohol consumption |  |  |  |  |
| Frequency, times/month | 1 (0 – 2) | 1 (0 – 2) | 1 (0 – 2) | -0.02 |
| Amount per occasion, glass/occasion | 1 (0 – 6) | 1 (0 – 6) | 1 (0 – 6) | -0.01 |
| Total alcohol intake, g/day |  |  |  | 0.02 |
| Systolic blood pressure, mmHg | 121 ± 19 | 120 ± 18 | 123 ± 20 | 0.09 |
| Diastolic blood pressure, mmHg | 80 ± 12 | 80 ± 12 | 81 ± 13 | 0.05 |
| Nutritional intake, g/day |  |  |  |  |
| Protein | 61 (48 – 78) | 62 (48 – 78) | 61 (47 – 79) | 0.00 |
| Fat | 28 (19 – 41) | 29 (19 – 41) | 28 (18 – 41) | -0.01 |
| Carbohydrate | 323 (278 – 385) | 325 (279 – 385) | 321 (275 – 384) | -0.01 |
| Sodium | 2.9 (2.1 – 3.9) | 2.9 (2.1 – 3.9) | 3.0 (2.1 – 4.0) | 0.03 |
| Laboratory variables |  |  |  |  |
| Hemoglobin, g/dL | 13.6 ± 1.6 | 13.6 ± 1.6 | 13.6 ± 1.6 | 0.00 |
| Serum albumin, g/dL | 4.5 (4.3 – 4.7) | 4.5 (4.3 – 4.7) | 4.5 (4.3 – 4.7) | -0.07 |
| Fasting blood sugar, mg/dL | 88 (82 – 95) | 88 (82 – 94) | 88 (82 – 96) | 0.05 |
| Total cholesterol, mg/dL | 189 (167 – 214) | 189 (168 – 213) | 189 (165 – 215) | 0.01 |
| AST, IU/L | 23 (19 – 28) | 22 (18 – 28) | 23 (19 – 29) | 0.07 |
| ALT, IU/L | 19 (14 – 28) | 19 (14 – 27) | 19 (14 – 28) | 0.02 |
| Serum creatinine, mg/dL | 0.8 (0.7 – 1.0) | 0.8 (0.7 – 0.9) | 0.8 (0.7 – 1.0) | 0.05 |
| eGFR, mL/min/1.73 m^2^ | 94 (81 – 104) | 95 (83 – 104) | 92 (80 – 103) | -0.10 |
| C-reactive protein, mg/L | 0.1 (0.1 – 0.3) | 0.1 (0.1 – 0.2) | 0.2 (0.1 – 0.3) | 0.05 |
| Albuminuria, % | 7 | 6 | 8 | 0.08 |

Note: values for categorical variables are shown as percentages; values for continuous variables, as mean ± standard deviation or median (interquartile range). ^*^Standardized difference is a difference in means or proportions divided by standard error; imbalance defined as absolute value greater than 0.20 (small effect size). ^**^Household income was categorized into three groups; <2,000,000 Korean won (KRW) per month (low), 2,000,000 to <4,000,000 KRW per month (middle), and ≥4,000,000 KRW per month (high).

Abbreviations: AST, aspartate aminotransferase; ALT, alanine aminotransferase; eGFR, estimated glomerular filtration rate.

**Supplemental Figure 1. Flow diagram of baseline recruitment and follow-up for the Korean genome and epidemiology study (KoGES).**


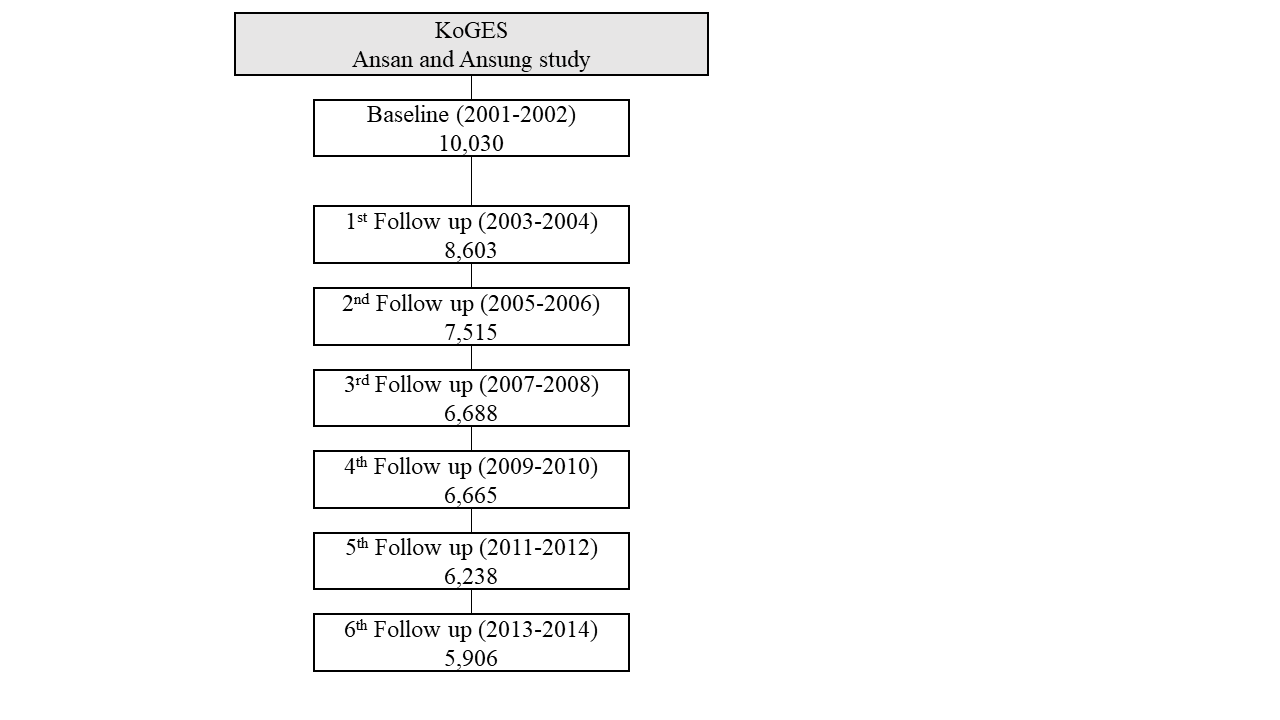


**Supplemental Figure 2. Cohort construction**


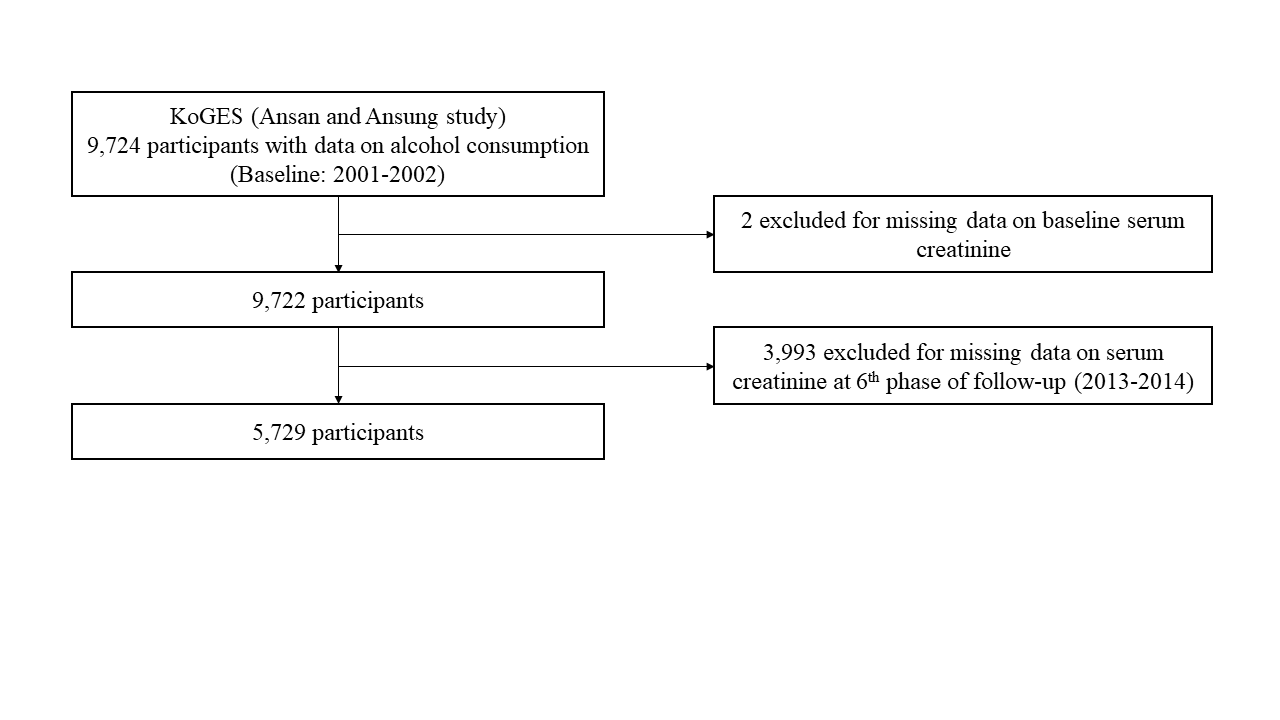


**Supplemental Figure 3. 12-year trajectories of total alcohol intake according to baseline alcohol consumption categories among 5,729 participants.**

**Supplemental Figure 4. The association of alcohol consumption with decline in kidney function over 12 years among 3,734 participants assigned to the same alcohol intake category as that at baseline at the last follow-up. Points and bars represent beta coefficients and 95% confidence intervals, respectively.**
